# Calcium channel signalling at neuronal endoplasmic reticulum-plasma membrane junctions

**DOI:** 10.1042/BST20230819

**Published:** 2024-06-27

**Authors:** Filip Maciąg, Arun Chhikara, Martin Heine

**Affiliations:** Institute of Developmental Biology and Neurobiology, Johannes Gutenberg University, Hanns-Dieter Hüsch Weg 15, 55128 Mainz, Germany

**Keywords:** endoplasmic reticulum, ER-PM junctions, membrane contact sites, ORAI, STIM, store-operated calcium entry

## Abstract

Neurons are highly specialised cells that need to relay information over long distances and integrate signals from thousands of synaptic inputs. The complexity of neuronal function is evident in the morphology of their plasma membrane (PM), by far the most intricate of all cell types. Yet, within the neuron lies an organelle whose architecture adds another level to this morphological sophistication — the endoplasmic reticulum (ER). Neuronal ER is abundant in the cell body and extends to distant axonal terminals and postsynaptic dendritic spines. It also adopts specialised structures like the spine apparatus in the postsynapse and the cisternal organelle in the axon initial segment. At membrane contact sites (MCSs) between the ER and the PM, the two membranes come in close proximity to create hubs of lipid exchange and Ca^2+^ signalling called ER-PM junctions. The development of electron and light microscopy techniques extended our knowledge on the physiological relevance of ER-PM MCSs. Equally important was the identification of ER and PM partners that interact in these junctions, most notably the STIM-ORAI and VAP-K_v_2.1 pairs. The physiological functions of ER-PM junctions in neurons are being increasingly explored, but their molecular composition and the role in the dynamics of Ca^2+^ signalling are less clear. This review aims to outline the current state of research on the topic of neuronal ER-PM contacts. Specifically, we will summarise the involvement of different classes of Ca^2+^ channels in these junctions, discuss their role in neuronal development and neuropathology and propose directions for further research.

## Introduction

**Figure 1.**
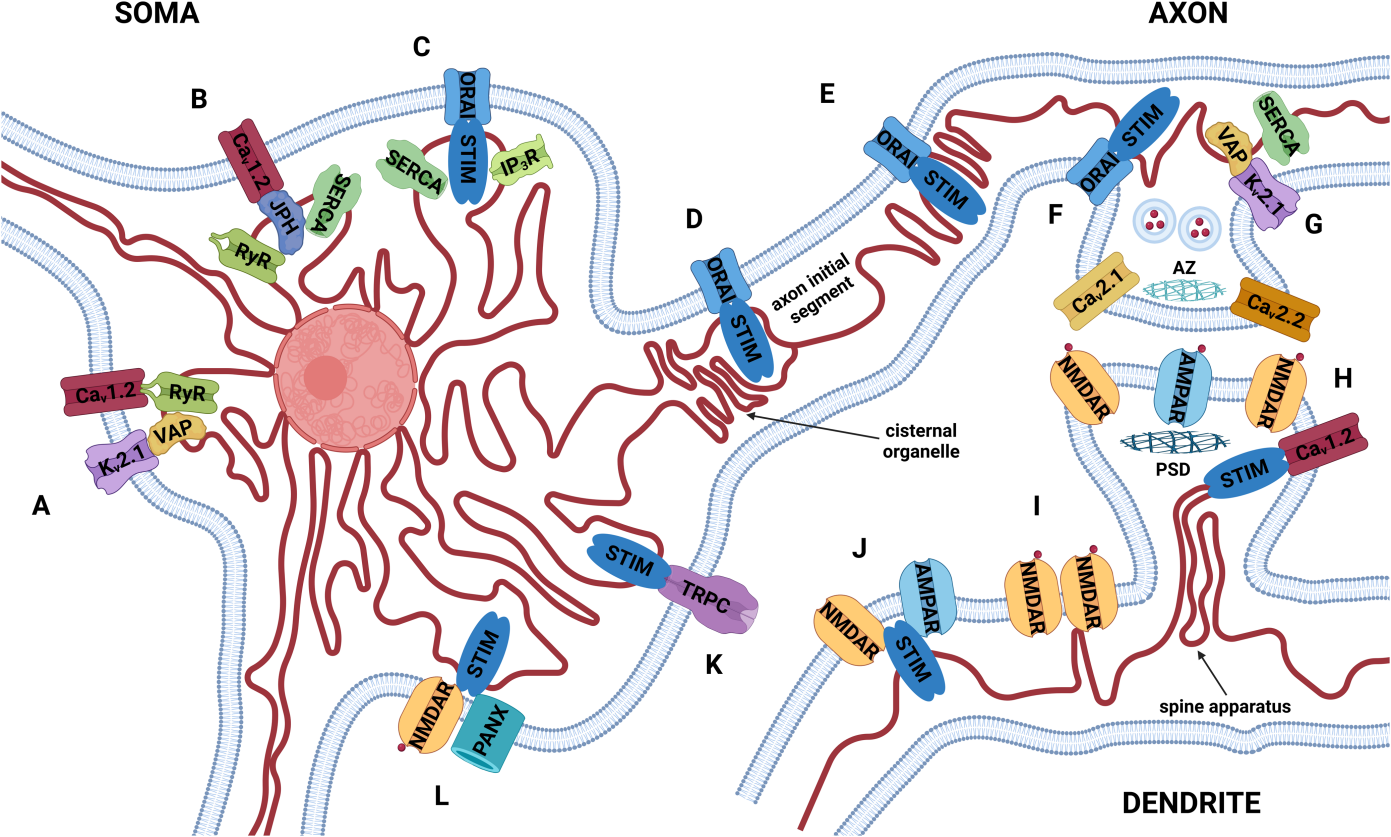
Overview of proteins that regulate Ca^2+^ signals in neuronal ER-PM junctions. The ER extends throughout the whole neuron, including the soma, dendrites and axons, and adopts specialised forms like the cisternal organelle and the spine apparatus. Notice that although ER-PM junctions are present in all neuronal compartments, the ER does not come in direct contact with the active zone (AZ) of neurotransmitter secretion or with the postsynaptic density (PSD) [1]. Letters denote the most important or newly identified ER-PM partners that are described in the main text. (**A**) Somatic plasma membrane K_v_2.1 channels cluster with VAP proteins in the ER and recruit Ca_v_1.2 and RyRs to ER-PM junctions to regulate excitation-transcription coupling [71,75,76]; (**B and C**) it has been suggested that VGCC-RyR and ORAI-IP_3_R pairs create two functionally independent ER Ca^2+^ pools [61]; (**D**) ORAI was implied to impact neuronal excitability [53–57]; (**E**) axonal ER in developing neurons was shown to adopt a ladder-like morphology, with STIM1 molecules colocalising with ORAI1 in structures termed ‘ER rungs’ [120]; (**F**) presynaptic STIMs were reported to play a role in neurotransmitter release via activation of ORAIs (by STIM2) [14] and by control of axonal ER Ca^2+^ stores (by STIM1) [15]; (**G**) VAP-K_v_2.1 clusters are implicated in activity-dependent uptake of Ca^2+^ to axonal ER, loss of K_v_2.1 impaired action-potential evoked Ca^2+^ influx into the presynapse and the release of neurotransmitter [16]; (**H**) STIM1 was shown to provide NMDAR-dependent feedback-inhibition of Ca_v_1.2 channels to limit excessive Ca^2+^ influx during glutamatergic neurotransmission [44]; (**I**) neuronal insults impact the morphology of ER-PM junctions: treatment with high doses of extracellular NMDA, which mimics glutamate spillover, significantly decreased the extent of ER-PM junctions [83], while nerve injury increased it [165]; (**J**) there is evidence for a direct interaction of STIM proteins with NMDA and AMPA receptors, impacting their activity and trafficking [39–43]; (**K**) TRPCs are a diverse family of proteins that can act as store-operated channels [45,46]; (**L**) pannexin has recently been identified as another store-operated channel; its activation is coupled to NMDARs and controlled by STIM1 [89]. Red dots represent glutamate. K_v_2.1, voltage-gated potassium channel 2.1; Ca_v_1.2, 2.1, 2.2, voltage-gated Ca^2+^ channels 1.2, 2.1, 2.2; VAP, VAMP-associated protein; RyR, ryanodine receptor; JPH, junctophilin; SERCA, sarco-/endopolasmic reticulum Ca^2+^ ATPase; STIM, stromal interaction molecule; IP_3_R, inositol triphosphate receptor; NMDAR, NMDA receptor; AMPAR, AMPA receptor; TRPC, transient receptor potential channel; PANX, pannexin; AZ, active zone; PSD, postsynaptic density. Figure created with BioRender®.

The endoplasmic reticulum (ER) is the biggest cellular organelle and plays critical functions such as protein and lipid synthesis. In neurons, the ER is present in the soma and extends to dendritic spines and axon terminals (Figure 1, [1]). Because of its intricate morphology and the crucial role as a Ca^2+^ store, it is capable of modulating neuronal function in all compartments. The ER can shape the Ca^2+^ homeostasis by generating Ca^2+^ signals via its own Ca^2+^ channels and pumps (inositol triphosphate receptors [IP_3_Rs], ryanodine receptors [RyRs], sarco-/ER Ca^2+^ ATPase [SERCA] and leak channels) or by modulating the function of plasma membrane (PM) Ca^2+^ channels. The latter is possible because of the presence of membrane contact sites (MCSs) between the ER and the PM, termed ER-PM junctions.

ER-PM junctions are specialised contact sites that are defined as areas of close apposition of the two membranes (∼10 nm in neurons, but the distance can reach hundreds of nm in non-excitable cells [2]). They are highly abundant in neurons, covering ∼10% of the PM [3], compared with 1–2% in non-excitable cells [2,4,5]. Multiple ER and PM proteins have been shown to localise to neuronal ER-PM contacts, including junctophilins (JPHs), vesicle-associated membrane protein (VAMP)-associated proteins (VAPs) and stromal interaction molecules (STIMs) in the ER membrane and voltage-gated potassium channels 2.1 (K_v_2.1) and ORAIs on the PM. The topic of MCSs has recently received considerable attention, in part due to advancements in methods that allow the examination of subcellular compartments at a nanometre scale [6–8]. These include cryo-electron tomography (cryo-ET), super-resolution light microscopy techniques, such as stimulated emission-depletion microscopy, structured-illumination microscopy, photoactivated localisation microscopy, total-internal reflection microscopy and others, and assays such as proximity ligation assay (PLA). These tools significantly expanded our understanding of neuronal ER-PM contacts. It is now well established that processes occurring in these junctions play critical roles in phenomena like neuronal excitability and plasticity (reviewed in [9]).

ER-PM junctions are most prominent on cell bodies, with fewer contacts present in dendritic and axonal branches [1]. Nonetheless, the ER has been shown to play direct roles in synaptic function. Already in the early 2000s, intracellular Ca^2+^ stores were reported to modulate neurotransmitter release [10–13]. These studies provided functional evidence for the role of ER in neurotransmission, but did not address the question whether ER impacts this process via direct contact sites with the PM. Because ER-PM junctions are excluded from active zones of transmitter secretion, it seems unlikely that these MCSs modulate neurotransmitter release by direct interaction with the vesicle fusion machinery (Figure 1, [1]). Nevertheless, research from the last decade has provided evidence for many presynaptic phenotypes for ER junctional proteins [14–16]. This progress in our understanding of ER actions in MCSs has been possible largely due to the discovery of molecular players responsible for store-operated calcium entry (SOCE) [17–22].

SOCE, earlier termed capacitative-calcium entry, is a ubiquitous mechanism that is of crucial importance in non-excitable cells, where it serves as the main pathway for Ca^2+^ influx from the extracellular space [23]. Because neurons are equipped with a variety of Ca^2+^ permeable channels, most notably *N*-methyl-d-aspartic acid receptors (NMDARs) and voltage-gated Ca^2+^ channels (VGCCs), there has been controversy regarding the physiological relevance of neuronal SOCE [24] and it is still unclear to which cellular processes it contributes to. Is it important for synaptic plasticity, homeostatic plasticity or excitability? In general, accumulating evidence suggests the role of SOCE and the proteins involved in this mechanism in neuronal function (see [25–31] for reviews). The molecules responsible for SOCE include STIMs, the Ca^2+^ sensors within the ER, and Ca^2+^ channels on the PM, most notably ORAI proteins. The STIM-ORAI system is a prime example of ER-PM signalling. Upon a drop in ER Ca^2+^ concentration, STIM proteins oligomerise and accumulate in ER-PM junctions, where they activate ORAI channels [32]. The resulting Ca^2+^ influx serves to replenish the ER stores and triggers intracellular signalling pathways. Both mammalian STIM isoforms, STIM1 and STIM2, have been suggested to affect neurotransmitter release, either by directly impacting presynaptic cytoplasmic [Ca^2+^] [14] or via control of axonal ER [Ca^2+^] [15]. The function of the presynapse was also shown to depend on a different ER-PM protein pair, namely clusters of PM voltage-gated potassium channels (K_v_2.1) and ER-resident VAMP-associated proteins (VAPs) [16,33,34]. Clusters of K_v_2.1 were shown to be indispensable for activity-dependent refilling of presynaptic ER Ca^2+^, with direct implications on vesicle fusion [16]. Despite these findings, our understanding of presynaptic ER actions is far from complete.

The ER also extends to postsynaptic sites, where it is estimated to be present in ∼40% of dendritic spines [35]. Here, the ER adopts a specialised form called the spine apparatus, which is suggested to play a role in synaptic plasticity [36]. Similarly as for the presynaptic side, ER-PM contacts are not expected to impact neurotransmission by direct interaction with the postsynaptic density (PSD), because they are localised away from the PSD (Figure 1, [1]). Nevertheless, different research groups reported a variety of ER-PM related postsynaptic effects, mainly involving the ER-resident STIM proteins. STIM1 and STIM2 have been suggested to differentially regulate dendritic Ca^2+^ signals [37], while lack of STIM2 prevented the maturation of mushroom spines [38]. STIM proteins have also been shown to impact the activity and trafficking of ionotropic glutamate receptors [39–43] and inhibit dendritic voltage-gated Ca_v_1.2 channels [44].

This review aims to summarise the most recent advancements in our understanding of neuronal ER-PM junctions with the focus on Ca^2+^ channels, particularly store-operated channels (SOCs), and their role in neuronal physiology, development and neurodegeneration.

## Store-operated Ca^2+^ channels

To date, two families of proteins have been established to function as SOCs: ORAI proteins and transient-receptor potential channels (TRPCs). Because the characterisation of neuronal TRPCs is beyond the scope of this review, the Reader is referred to other works on this topic [45,46], while this review will focus on the role of neuronal ORAIs. ORAI proteins (ORAI1, 2 and 3 isoforms in mammals) form hexameric Ca^2+^-release activated channels, whose hallmarks are a high selectivity to Ca^2+^ ions and small conductance [47]. To the best of our knowledge, there have been no reports of ORAI activation independent of ER-resident STIM proteins in neurons (although in non-excitable cells, ORAI1 and ORAI3 build store-independent arachidonate-regulated Ca^2+^ channels [ARC channels] that are activated by PM-resident STIM1 [48,49]). Therefore, it is valid to assume that the only sites of neuronal ORAI-mediated Ca^2+^ entry are ER-PM junctions. Complexes of endogenous neuronal STIM1 and STIM2 with ORAI1 were first shown by the Kuźnicki group by PLA [50]. Here, positive PLA signals were found mostly on cell bodies and only occasionally in neuronal processes. In later studies, ORAI was reported to be present in dendritic spines of cultured rat hippocampal neurons, where its role in long-term potentiation (LTP) and spine formation was proposed [51,52]. The same group reported that Ca^2+^ influx via ORAI1 underlies the impact of ER Ca^2+^ stores on neuronal excitability [53]. Evidence for the role of ORAI in excitability also comes from studies on animal models: rodents, in the context of epilepsy and nociception [54–56], and the fruit fly, where Ca^2+^ influx through ORAIs impacted the expression of excitability-related genes [57].

Recently, a link between ORAI and NMDAR was identified: knockout of ORAI1 significantly diminished NMDAR-dependent postsynaptic Ca^2+^ signals, impacting LTP of hippocampal CA1 neurons [58]. Whether NMDARs themselves are present in ORAI-containing ER-PM junctions was not investigated in this study. The aforementioned work sparked an intriguing controversy, whereby an independent research group highlighted the importance of ORAI2, but not ORAI1, in shaping Ca^2+^ signals in CA1 hippocampal neurons [59,60]. Of note, the latter group suggested the existence of two functionally distinct ER Ca^2+^ pools in these cells, one dependent on the ORAI2-IP_3_R pair and the other on the VGCC-RyR dyad [61,62]. This concept supports the idea that neurons possess different classes of ER-PM junctions that independently control distinct ER Ca^2+^ pools.

The coupling between the IP_3_R and SOCE was first established by the use of HEK cells as a model [63]. Here, it was shown that activation of IP_3_Rs via IP_3_ leads to depletion of ER Ca^2+^ and induction of a store-operated current via TRP channels. More recently, an intricate relationship between IP_3_Rs and all five STIM and ORAI isoforms was described, with implications on spontaneous cytosolic Ca^2+^ oscillations in HEK cells [64]. Evidence for the role of neuronal IP_3_Rs in ER-PM junctions comes from the Hasan laboratory, who showed that in Drosophila neurons, migration of IP_3_Rs to ER-PM contact sites promotes STIM-ORAI coupling and consequently SOCE [65]. Interestingly, recent data from this group suggest that IP_3_Rs impact SOCE independently of their canonical function as Ca^2+^ channels [66].

The studies mentioned so far used the classical Ca^2+^ — addback protocol or induction of synaptic activity to study the role of ORAIs in neurons. Of note, the presence of store-operated Ca^2+^ signals was also reported in dendritic spines in the absence of synaptic activity, suggesting that ORAI channels are stochastically active in the postsynapse [67]. Interestingly, ORAI-dependent ER store replenishment was shown to occur more prominently in the spine head, while RyR-dependent Ca^2+^-induced Ca^2+^ release (CICR) in the spine base. The authors propose an elegant mechanism, whereby spatial isolation of ORAI-SERCA pair and RyR clusters conveys a functional separation of the two ER-dependent Ca^2+^ signalling modes: Ca^2+^ replenishment (ER as a Ca^2+^ sink, ORAI-SERCA) and Ca^2+^ release (ER as a Ca^2+^ source, RyR). Future studies are expected to shed more light on the involvement of ORAI in synaptic function in both the pre- and the postsynapse.

### Voltage-gated Ca^2+^ channels

VGCCs play a pivotal role in neuronal signalling and their function is critically determined by subcellular localisation. For instance, clustering of VGCCs in the presynaptic active zone is essential for their well-studied role in neurotransmitter release [68,69]. The localisation of VGCCs in relation to the ER had been an unexplored field until recently, when membrane proteins K_v_2.1 and JPHs were shown to facilitate VGCC clustering at ER-PM junctions (reviewed in [70]). In hippocampal neurons, K_v_2.1 promotes the clustering of somatic Ca_v_1.2, altering their kinetics and enhancing their activity [71]. Several studies have shown a connection between VGCC, Ca^2+^ sensitive potassium channels and RyRs [72,73]. Sahu et al. identified a complex of RyRs, Ca_v_1 and K_Ca_3.1, tethered by ER-resident JPHs, which controlled excitability of hippocampal neurons. This work was expanded by studies of Perni and Beam, who used a heterologous expression system to show that JPHs promote RyR localisation in ER-PM contacts in an isoform-specific manner, and that they modulate the function of VGCCs [74]. Functionally, RyRs that are present in ER-PM junctions organised by the K_v_2.1 — VAP pair were shown to control excitation-transcription coupling in rat hippocampal neurons [71,75,76]. For a thorough description of the role of RyR and VGCC coupling in ER-PM junctions, the Reader is referred to a recent excellent review [70].

In contrast with the stimulatory effect of K_v_2.1 on intracellular Ca^2+^ signals, STIM proteins were shown to act as inhibitors of Ca_v_1.2 [77,78]. In [44], the researchers proposed an elegant mechanism by which glutamatergic transmission depolarised the postsynaptic membrane, triggering the opening of Ca_v_1.2 and leading to CICR, subsequently causing activation of STIM1 molecules that feedback-inhibited Ca_v_1.2 channels. While STIMs, JPHs and K_v_2.1 have all been shown to impact the distribution and activity of VGCCs, it is unlikely that they populate the same ER-PM junctions at a given time point. VAP, the ER partner of K_v_2.1, localises specifically to K_v_2.1 junctions, but not to those mediated by JPHs [34]. Moreover, glutamate has been shown to dissolve clusters of K_v_2.1 [79], but induction of CICR causes clustering of STIM molecules [44].

Recently, compartmentalisation of ER voltage signals has been reported [80]. With the use of a novel genetically-coded fluorescent voltage indicator targeted to the ER, ASAP3_ER_, the authors demonstrated that stimulation of both RyR and IP_3_R results in a voltage change across the ER in the range of tens of millivolts. Importantly, these signals were spatially restricted by the activity of PM BK channels. This adds another factor to the complexity of ER-PM communication and opens the possibility that ER voltage signals directly impact the function of VGCCs that are localised to ER-PM junctions, e.g. to modulate neuronal excitability.

### Ligand gated Ca^2+^ channels

Research from the last two decades provided evidence that the two most widely studied ionotropic glutamate receptors, NMDARs and AMPARs, participate in signalling in ER-PM junctions. Glutamate stimulation was shown to dissolve VAP- and K_v_2.1-dependent ER-PM junctions via Ca^2+^ — dependent dephosphorylation [79,81,82]. A decrease in the extent of ER-PM contacts upon NMDAR stimulation was later confirmed with electron-microscopy studies [83]. Similarly to K_v_2.1 channels, pro-neuregulin-2 (ProNRG2), an epidermal-growth factor-like ligand of ErbB3/4 receptor tyrosine kinases, also acts as an activity-dependent organiser of ER-PM junctions, by binding to VAPs on the ER membrane [84,85]. Here, the authors showed that NMDARs activation led to dephosphorylation of ProNRG2, disrupting its interaction with VAPs and causing dissociation from ER-PM junctions. Additionally, NMDAR-induced Ca^2+^ influx alters the lipid composition of ER-PM junctions by facilitating the recruitment of the lipid transporter TMEM24 to these contacts [86]. Whereas the aforementioned publications highlight the impact of NMDAR activity on the integrity and composition of the ER-PM contacts, it is important to acknowledge the reciprocal effect on NMDARs. While the recruitment and removal of NMDARs from the PM depend on Ca^2+^ flux and their phosphorylation status, the lipid composition of ER-PM junctions also influences their retention [87,88]. Weesner et al. [87] proposed that GM1-ganglioside promotes the retention of active, phosphorylated NMDARs in ER-PM junctions, which in turn activate Ca^2+^-mediated ERK signalling and facilitate dendritic spine formation.

The putative localisation of NMDARs at ER-PM junctions positions them strategically for potential interaction with STIM proteins. Using immunoprecipitation, Gruszczynska-Biegała et al. [40] demonstrated that STIM1 and STIM2 interacted with the NMDAR subunits GluN2A/B in rat cortical neurons. Activated STIMs were proposed to negatively regulate the Ca^2+^ influx via NMDARs. The same group demonstrated that STIM2 promoted internalisation GluN2A/B subunits after their overactivation [41]. A recent study provided evidence that pannexins, proteins that build nonselective PM channels, could be activated in an NMDAR- and STIM1-dependent manner [89]. This work identified pannexins as another SOC, with potential implications for the role of ER-PM junctions in coordinating neuronal communication between chemical and electrical synapse [90,91].

Although NMDARs and VGCCs are regarded as the main contributors to neuronal Ca^2+^ signals, a subset of AMPA receptors, particularly GluA2 lacking AMPARs, are permeable to Ca^2+^. Accumulating data suggest vital roles for Ca^2+^-permeable AMPARs in physiology and disease [92–94]. Importantly, AMPARs were shown to colocalise with STIM proteins and to directly contribute to Ca^2+^ influx via SOCE [39]. The notion that AMPARs shape Ca^2+^ signals in ER-PM junctions is strengthened by a recent finding that extended synaptotagmins (E-Syts), a family of ER junctional proteins, play a role in LTP-induced increase in surface expression of AMPARs [95]. In earlier work [42], STIM2 was shown to promote phosphorylation of GluA1 subunits, likely in extra-synaptic ER-PM junctions. Knockout of STIM2 resulted in impaired LTP and dendritic spine morphology, highlighting the importance of ER-PM Ca^2+^ signalling in synaptic plasticity [43].

### ER-PM calcium signalling in neuronal development

One of the main challenges that neurons face during development is providing the substrates for the rapidly growing axon [96]. The ER, with its capacity of lipid synthesis and transport, provides a platform to supply the PM with lipids during growth [97]. Indeed, many lipid transfer proteins bind to VAPs, ER-PM membrane tethers that are crucial for neuronal physiology [98,99]. A recent study identified the presence of VAP oligomers in neurites of young (7 DIV), but not in more mature (14 DIV) dendrites of mouse hippocampal neurons [100]. Interestingly, the abundance of K_v_2.1 clusters, which colocalise with VAPs in neuronal ER-PM junctions [33,34], increases with *in vitro* ageing [101]. K_v_2.1 and VAPs were recently shown to colocalise with a lipid transporter TMEM24/C2CD2L, which is also found in neuronal ER-PM junctions and whose expression increases with age [86]. Both K_v_2.1 and TMEM24 clusters depend on Ca^2+^, in that the influx of Ca^2+^ via NMDARs alters the phosphorylation state of the two proteins, leading to dissolution of K_v_2.1 and TMEM24 clusters [79,86]. These studies suggest that ER-PM MCS are remodelled during neuronal development in Ca^2+^ — dependent processes.

A recent work demonstrated that STIM1 is enriched in the spine apparatus [102], an ER structure that is predominantly found in mature spines [103]. Interestingly, lack of STIM2, but not STIM1, was reported to impair the formation of mushroom spines in mouse hippocampal neuronal culture, with implications in the pathology of Alzheimer's disease (AD) [38,104]. Kushnireva et al. [37] suggested that STIM1 and STIM2 play differential roles in mouse hippocampal dendritic spines, with STIM1 being expressed most prominently in developing spines, while STIM2 localising mostly to mature ones. In this study, it was also observed that STIM puncta were mobile along the dendrite and their movement correlated with Ca^2+^ sparks, suggesting occasional visits of STIM molecules to ER-PM junctions populated by SOCs. The notion that Ca^2+^ influx through SOCs can play a role in neuronal development is strengthened by an earlier work, where the authors reported a higher amplitude of SOCE in 4–8 DIV neurons, compared with 15 DIV [105]. In the same study, older neurons were shown to have a higher concentration of ER Ca^2+^.

Age-related changes in ER Ca^2+^ have been demonstrated by multiple research groups and support the so-called Ca^2+^-hypothesis of ageing [106–109], but the potential impact of ER stores on neuronal development is less well characterised. ER Ca^2+^ has been shown to contribute to dendritic NMDA-dependent Ca^2+^ signals in young neurons [110], but this effect appeared to be less prominent, or even negligible, in mature cells [111–114]. More recently, CICR that occurred exclusively in developing dendritic spines was shown to prolong NMDAR-dependent dendritic Ca^2+^ signals and drive local cooperative plasticity along the dendrites of mouse CA1 pyramidal neurons [115]. The potential contribution of ORAI channels was not discussed in this work. Notably, studies from a different research group suggest that deletion of ORAI1 significantly affects LTP, but has no impact on spine density in mouse CA1 pyramidal neurons [58]. Further investigations are needed to clarify what is the role of ORAI in synaptic development and homeostasis.

Recent data has provided evidence that cross-talk between ER-resident proteins and microtubules is crucial for the establishment of neuronal polarity [116]. In rat DRG neurons, STIM1 and ORAI1 were shown to preferentially localise towards neuronal growth cone and knockdown of STIM1 interfered with growth cone turning in response to physiological cues [117]. STIM1 knockdown also abolished brain-derived neurotrophic factor — induced SOCE in growth cones, suggesting that STIM-ORAI signalling is important for axonal development. The role of SOCE in neurite outgrowth was also confirmed in *Xenopus* spinal cord neuron growth cones [118] and in differentiated rat PC-12 cells [119]. Whether ER-PM MCSs undergo dynamic changes during development of axonal ER remains an outstanding question. Yet, recent data provide evidence that the morphology of mature axonal ER significantly differs from that of a developing neuron [120]. In 3–7 DIV, but not 18–21 DIV mouse hippocampal neurons, the ER adopted a characteristic form that the authors termed the ‘ER ladder’, with ER ‘rails’ running along microtubule bundles, and ER ‘rungs’ arranged perpendicularly. Of note, depletion of ER Ca^2+^ caused STIM1 to accumulate in ER ‘rungs’, in puncta that were co-localised with ORAI1. This highlights the notion that Ca^2+^ channel signalling in neuronal ER-PM MCSs plays a role in the maturation of axonal ER. What exactly is the function of ORAI-dependent Ca^2+^ signals in this context remains to be discovered.

### ER-PM calcium signalling in neurodegeneration

A number of studies have linked neurodegenerative processes with the altered functions of the ER. These include disturbances in the activity of ER Ca^2+^ channels [106,121–125] and changes in the structure of the ER (reviewed in [126]). Despite the high abundance of ER-PM contacts in neurons, surprisingly little is known about the involvement of these MCSs in neurodegenerative processes. Indirect evidence comes from the links between mutations in genes coding junctional proteins and neurological disorders. A repeat expansion in *JPH3* is implicated in Huntington's disease (HD)-like 2 [127], while nucleotide variants of *JPH1* were linked to a Charcot–Marie–Tooth disease [128,129]. Loss-of-function mutations in *VAP-B* cause a rare form of amyotrophic lateral sclerosis, ALS8 [130,131]. VAP-A and its interaction with K_v_2.1 have been implicated in the pathology of ischaemic injury [132], whereas VAP-B is involved in the pathology of Parkinson's disease and multiple system atrophy [133–135]. Because both VAPs and JPHs have been shown to cluster in the proximity of ER and PM Ca^2+^ channels, it is plausible to assume that altered Ca^2+^ homeostasis in ER-PM junctions plays a role in these neurodegenerative phenotypes.

In spite of the paucity of studies that directly address the subject of ER-PM contact sites in the context of neuropathology, there is now ample evidence for the involvement of SOCE signalling in neurodegenerative diseases (reviewed in [28,126]). Alterations in expression of STIM2 were reported in lymphocytes from familial AD (fAD) patients [136] and later confirmed in presenilin-knock-in hippocampal neurons, a model of fAD [38]. Moreover, a down-regulation of STIM2 expression was found in lysates of cortical samples from sporadic AD patients, and the level of STIM2 positively correlated with the patients' score in mini-mental status test [38]. Moreover, a complex of ORAI2, TRPC6 and STIM2 was identified as a potential target to combat AD-related memory loss [137,138]. Later studies suggested that also STIM1 plays a role in the disturbed neuronal SOCE in a model of fAD [139]. A link between SOCE and fAD has recently been confirmed in a study which showed that signalling via SOCs and mGluRs contributes to the dysregulation of Ca^2+^ homeostasis seen in mouse fAD models [140]. Moreover, multiple disturbances in the function of ER Ca^2+^ release channels, IP_3_Rs and RyRs, have been linked to AD (reviewed in [141–144]).

Alterations in ER-PM signalling have also been reported to underlie the pathology of (HD, reviewed in [145]). In early 2000s, mutated huntingin (mHTT) and huntingtin-associated protein 1 were shown to sensitise IP_3_R to activation by IP_3_ [122,123]. Subsequently, increased Ca^2+^ influx via SOCE was reported in medium spiny neurons isolated from YAC128 mice, a model of HD [146–148], as well as in patient-derived induced pluripotent stem cells [149,150]. Excessive SOCE caused synaptic loss characteristic of HD [151] and could be prevented by knockdown of ORAI1/2 and TRPC1/6 [152]. These works provided first indirect evidence for the involvement of mHTT in ER-PM Ca^2+^ signalling. Notably, recent data obtained from skeletal muscle suggest that mHTT may directly act at ER-PM junctions via an interaction with JPH1 [153]. Whether such mechanism takes place also in neurons remains an open question.

The activity of SOCs has also been implicated in the pathology of epilepsy. Increased levels of STIM1 and STIM2 were found in hippocampi of temporal lobe epilepsy patients and inhibition of SOCE normalised the activity of chronically epileptic hippocampal slices [154]. Hippocampal preparations isolated from mice with neuronal overexpression of ORAI1 showed changes in electrical activity upon stimulation with pro-epileptic drugs [54]. Interestingly, aged female mice used in this study developed spontaneous seizures, suggesting sex-specific differences in SOCE signalling. Subsequent RNAseq analysis of hippocampi isolated from these mice showed changes in the expression of genes that are implicated in rare epilepsy-associated disorders [155]. Soon thereafter, ORAI1 was shown to control the excitability of mouse hippocampal GABAergic neurons, whereby loss of ORAI1 sensitised the animals to chemiconvulsant-induced epileptic seizures [56]. Altogether, these studies provide solid evidence for the role of ORAI1-mediated ER-PM signalling in neuronal excitability in the context of epilepsy.

ER-PM signalling is implicated not only in chronic, but also in acute neuropathology. Berna-Erro et al. established STIM2 as a basal regulator of neuronal Ca^2+^ level and suggested the role of STIM2 in neuronal death during focal-cerebral ischaemia [156]. A later work from this group identified ORAI2 as the STIM2-activated channel that provided excessive Ca^2+^ in ischaemic conditions [157]. Interestingly, two other research groups proposed that it is the STIM1-ORAI1 mediated SOCE that contributes to ischaemia-related neuronal loss [158,159]. This discrepancy in STIM-ORAI isoforms could lie in the different experimental models used by the researchers (mouse in the former, and rat in the two latter groups).

Interestingly, blockade of SOCE prevented autophagic processes triggered by ischaemia-modelling treatments [160]. Autophagy of the ER, termed ER-phagy, is an exciting chapter in the field of neurodegeneration [161]. Of note, loss of autophagy related 5 protein (ATG5) was shown to trigger accumulation of tubular ER in the axon, resulting in higher density of RyRs and consequently facilitation of neurotransmission [162]. Autophagic processes are suggested to play a role in neuronal injury, although their exact contribution to trauma-related pathophysiology of the neuron is poorly understood [163,164]. Notably, the extent of ER-PM contacts was reported to significantly increase following injury [165], and there is substantial evidence suggesting the involvement of ER-PM communication in the formation of autophagosome [9,166]. The yet undiscovered link between autophagic processes and Ca^2+^ signalling at ER-PM junctions is an intriguing avenue of future studies.

## Perspectives

- ER-PM communication is increasingly recognised as an important factor that shapes neuronal physiology. Recent advancements in super-resolution microscopy and cryo-ET allowed us to broaden our understanding how Ca^2+^ signals generated at ER-PM junctions impact neuronal function.
- Some of the outstanding questions include: are there different classes of ER-PM junctions that are regulated by spatially and functionally separated ER Ca^2+^ pools? Will the family of SOC channels expand beyond ORAI and TRPC proteins and include e.g. pannexins? What is the role of ER-PM junctions in ER-phagy? How stable are these junctions? What is the turnover of their molecular composition?
- Recent studies suggest that dynamic changes in ER-PM junctions occur during development and neurodegenerative processes. Deciphering their nature is expected to translate into clinically relevant therapeutic approaches.

## Abbreviations

AD: Alzheimer's disease
CICR: Ca^2+^-induced Ca^2+^ release
cryo-ET: cryo-electron tomography
ER: endoplasmic reticulum
fAD: familial AD
HD: Huntington's disease
JPH: junctophilin
LTP: long-term potentiation
MCS: membrane contact site
mHTT: mutated huntingin
NMDAR: *N*-methyl-d-aspartic acid receptor
PLA: proximity ligation assay
PM: plasma membrane
PSD: postsynaptic density
SOCE: store-operated calcium entry
SOC: store-operated channel
STIM: stromal interaction molecule
TRPC: transient-receptor potential channel
VAMP: vesicle-associated membrane protein
VGCC: voltage-gated Ca^2+^ channel
